# Mathematical Modelling, Simulation and Optimisation of Microneedles for Transdermal Drug Delivery: Trends and Progress

**DOI:** 10.3390/pharmaceutics12080693

**Published:** 2020-07-22

**Authors:** Prateek Ranjan Yadav, Tao Han, Ololade Olatunji, Sudip K. Pattanayek, Diganta Bhusan Das

**Affiliations:** 1 Chemical Engineering Department, Loughborough University, Loughborough LE11 3TU, Leicestershire, UK; P.R.Yadav@lboro.ac.uk (P.R.Y.); hantaotony@gmail.com (T.H.); 2 Chemical Engineering Department, Indian Institute of Technology, Delhi 110016, India; sudip@chemical.iitd.ac.in; 3 Department of Chemical and Petroleum Engineering, University of Lagos, Lagos 100213, Nigeria; lolakinola@gmail.com

**Keywords:** microneedles, skin, transdermal, drug delivery, modelling, optimisation

## Abstract

In the last two decades, microneedles (MNs) have received significant interest due to their potential for painless transdermal drug delivery (TDD) and minimal skin damage. MNs have found applications in a range of research and development areas in drug delivery. They have been prepared using a variety of materials and fabrication techniques resulting in MN arrays with different dimensions, shapes, and geometries for delivery of a variety of drug molecules. These parameters play crucial roles in determining the drug release profiles from the MNs. Developing mathematical modelling, simulation, and optimisation techniques is vital to achieving the desired MN performances. These will then be helpful for pharmaceutical and biotechnological industries as well as professionals working in the field of regulatory affairs focusing on MN based TDD systems. This is because modelling has a great potential to reduce the financial and time cost of both the MNs’ studies and manufacturing. For example, a number of robust mathematical models for predicting the performance of the MNs in vivo have emerged recently which incorporate the roles of the structural and mechanical properties of the skin. In addressing these points, this review paper aims to highlight the current status of the MN modelling research, in particular, the modelling, simulation and optimisation of the systems for drug delivery. The theoretical basis for the simulation of MN enhanced diffusion is discussed within this paper. Thus, this review paper provides a better understanding of the modelling of the MN mediated drug delivery process.

## 1. Introduction

The microneedle (MN) system has received significant interest in the last two decades as an important method for transdermal drug delivery (TDD). The MNs consist of micron size needles that facilitate drug molecules to overcome the stratum corneum (SC), the outermost layer of skin, without triggering the nerve ending within the dermis [1,2]. Thus, painful events such as in the case of applying a hypodermic needle can be averted [3]. Since the first demonstration of the applicability of MN in 1998 [1], it has been recognised as an alternative to traditional methods for the delivery of vaccines, drugs and cosmetics [4]. For example, MNs have been employed recently to improve the transdermal permeation of insulin [5], caffeine [6], human growth hormone [7], lidocaine [8], ovalbumin [9], bovine albumin [10], bevacizumab [11], rapamycin [12], Calcein [13], parathyroid hormone [14] and levonorgestrel [15]. MNs have also been explored for the delivery of other molecules such as peptides, ocular drugs and cancer drugs [16].

**Figure 1:**
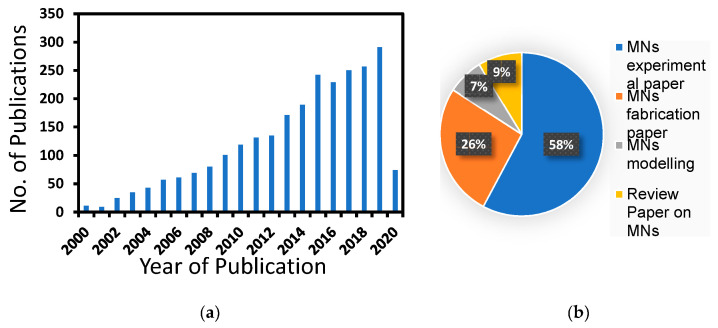
(**a**) Number of journal articles published related to microneedles (MNs) according to Scopus in between 2000–2020, (**b**) distribution of papers according to different MN applications (accessed on 17 March, 2020).

As shown in Figure 1a, the publications related to MNs have grown in the last two decades at an impressive rate. It seems that the reported MN research is mainly focused on two broad areas: (i) MN-based process developments and (ii) MN fabrication strategies. Under (i), laboratory-based experimental studies of various molecules, vaccines and microparticles delivery through the MN and skin have been conducted [17,18,19]. While under (ii), various technologies such as micro-electromechanical systems (MEMS) [20,21], laser cutting [22,23], 3D printing [24,25], and lithography [26,27,28] have been applied for production of MN.

Mathematical modelling of the MN systems is an area of growing research where the theories governing drug transport and delivery through the skin can be suitably applied for the development and optimisation of MN systems. However, it is obvious that most of the research in the field of MN is focused towards an aspect of experimental or/and fabrication technique. The attention towards the modelling has been ignored generally as researchers tend to focus more on characterisation and fabrication of the MNs. The exact reason for a significantly less efforts on development of modelling, simulation and optimisation tools for MNs is not known. We believe this is due to a lack of sufficient number of parameters for applying the relevant theories as well as the expertise and interests needed for modelling these systems. An analysis of these publications is shown in Figure 1b, which confirms this observation. In fact, the small number of publications related to modelling of MN systems (7%) suggests that the attention in this regard is insufficient at the moment. Focusing more research efforts toward the prediction of MN performance via modelling should help direct the overall research effort towards critical areas that need addressing within the MN research and development. A review of the existing mathematical models is, therefore, essential to bring together the work done so far on MNs and, identify, the key areas which need further studies.

**Figure 2:**
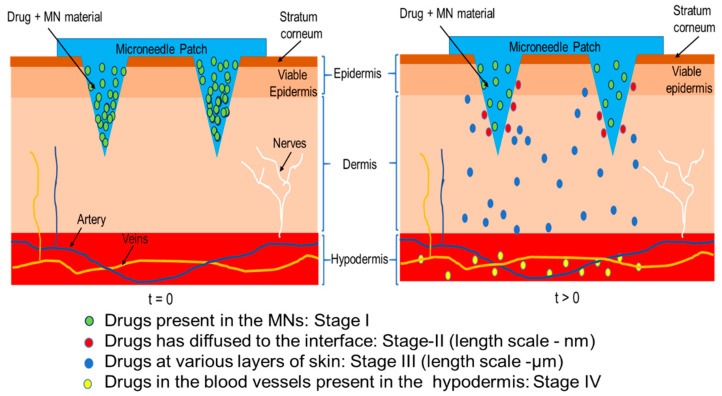
Schematic of drug transport in MN treated skin.

The possibility of developing modelling tools towards achieving MN optimisation and testing in support of experimental studies is beneficial and attractive. It can save both the costs and time by eliminating the need for many time-consuming and expensive experimental trials. Modelling MN based TDD typically deals with the simulation of three coupled sub-domains i.e., MNs, skin, and bloodstream. As shown in Figure 2, the drugs diffuse from the individual MN to the epidermis and/or dermis region of skin and subsequently reach the bloodstream. Figure 2 also shows the length scale and stages of drug transports in the MN. The differences in drug delivery behavior within the skin is due to the difference in the properties of different layers of skin.

Keeping the above points in mind, this review aims to present the current status of various modelling approaches for MN design and their utilities. This article also highlights the ways in which skin properties can be incorporated in the modelling of MN based TDD. For completeness of the discussion in this paper, we will briefly discuss the key features of the MNs, and the parameters required for carrying out mathematical modelling of these systems. Published case studies on various types of MNs are also highlighted in the latter part of the paper. We believe that this is the first review paper that entirely focuses on reviewing the state of the published work on mathematical modelling, simulation and optimisation of MNs.

## 2. MN Features

Some understanding of the MN features is necessary as the first step in the modelling process. This is because reasonable design criteria are necessary to guarantee that a MN array is functioning well during drug delivery [29] or extracting interstitial skin fluid [30]. Previous researchers have come up with a variety of MN types for effective drug permeation and transport in the skin. Various materials have also been used to fabricate these MNs, e.g., metals, polymers, and others. These are all key information for a modelling, simulation and optimisation tool. We will, therefore, discuss these key features of MNs in brief, and in particular, the different types of MNs developed so far and materials used for MN fabrication. We discuss various designs of MNs investigated by the researcher for efficient drug delivery into the skin. As these issues have been discussed in earlier papers (e.g., [2]), they are only discussed briefly in this section so as to ensure completeness of the paper as well as to facilitate the discussion better in the latter part of the paper.

### 2.1. Morphological Variation of MNs

Morphologically, MNs are categorised into five types, namely, hollow, solid, coated, dissolving, and swellable MNs [31]. As discussed in numerous reviews [3,4,32] on the topic, the MN types must be properly chosen according to their applications. Hollow MNs perform in the same way as regular hypodermic needles but have much shorter capillaries within them. The chosen liquid drug formulation is pressurised through micron-size holes in these MNs [33]. The hollow MNs are also applicable for fluid extraction [34,35] and solute monitoring [36,37]. However, their usage is limited by inherent structural weakness due to the thin walls and fabrication complexities. Solid MNs have high material stiffness and more stable structures than the hollow MNs and, they can penetrate the skin more effectively than hollow MNs [38]. Solid MNs are penetrated into the skin, which are then detached with a view to creating micron size channels in the skin. A transdermal patch or micro-emulsion of drug is then applied creating a reservoir for TDD [8,39,40,41]. This type of MNs requires a two-step application, which may make them less convenient for patients. The appearances of coated MNs, which are coated with a drug formulation on the MN surfaces, has resolved this problem. After the application of drug coated MNs in the skin, the coating is dissolved in the skin [42,43,44]. However, the drug loading amount is relatively low in coated MNs, which limit their applications in the case of high dose delivery [45]. The dissolving MNs are designed to be more suitable for the delivery of rapid/controlled release formulations, or in situ-forming implants [46,47]. It is made up of polymers and can deliver large molecules, micro-particles, or vaccines. They have the capacity to load larger amounts of drug in the polymeric matrix. The foundations of the swellable MN mechanism involves the absorption of local moisture within the skin and opening up the pore-space within the polymeric matrix, enabling the delivery of the drug from the needle into the skin by diffusion [48,49]. The swellable needle patch can be removed after the drug delivery process is complete. This reduces the risk of infection and skin irritation due to polymer dissolution in the skin [50].

However, there are still withstanding issues, such as the manufacturing cost and complexities, toxicity, and the physicochemical properties of the drug molecules that limit the range of applications needing further attention [51,52,53,54]. Thus, from the above discussion, it is evident that each MN has significant merits and demerits in terms of its applications to drug delivery. MN modelling and optimisation tools have the potential to screen the appropriate choice of a MN type in a given case.

### 2.2. Materials of MN

Of the key MN features, namely, the material of fabrication of the MNs is a critical factor in the preparation and application of different kinds of MNs. Different designs of MNs require specific properties of materials for preparation. These aspects become important for developing mathematical models of these systems as well. Silicon is known to be the material of the first MNs used for drug delivery due to the advent of MEMS technology during the 1990s [1,55,56]. Silicon or glass provides significant flexibility in MN fabrication processes that can be used to shape them and can be micro-structured in a variety of desirable shapes and sizes. However, various metals (e.g., stainless steel) have also been used widely in preparing solid MNs due to their good mechanical strengths. These MNs overcome the skin barrier functions and enhance drug permeability. The MN that have been prepared using these materials are: (i) solid [57,58], (ii) hollow [59,60] and (iii) coated MNs [61]. However, the above MNs suffer from various disadvantages like skin irritation and expensive fabrication processes [62]. For example, there is a possibility that the glass MNs may break in the skin and cause safety concerns [63].

The limitations of metal and inorganic MNs such as expensive production processes and bio-incompatibility have led to the development of polymeric MNs. These polymers can be used to prepare solid [13], coated [64], dissolving [65] and hollow MNs [66]. Polymeric MNs are easy to fabricate on larger scales and can be loaded with the chosen drug on the MN tips and/or base or they can be coated on the MN tips with high loading amounts. Depending on how they perform, the MNs can be grouped into dissolvable, swellable and biodegradable MNs. The polymers for the MNs have excellent biocompatibility, biodegradability, low toxicity, strength/toughness and low cost. They include poly(methyl methacrylate) (PMMA), poly-L-lactic acid (PLA), poly-glycolic acid (PGA), polylactide-co-glycolide acid (PLGA), poly(carbonate), cyclic olefin copolymer, poly(vinylpyrrolidone) (PVP), poly(vinyl alcohol) (PVA), polystyrene (PS), poly(methyl vinyl ether-co-maleic anhydride) (PMVE/MA), SU-8 photoresist, polysaccharides and others. Details about these polymers and their use have been studied in other review papers [19,31] and are not discussed in detail in this paper.

With the development of dissolving MNs, researchers have started to encapsulate drugs and vaccines in polymers for TDD, which make the biocompatibility of these polymers a top priority for their design [67,68]. MNs have been produced from biopolymers such as silk fibroin proteins [69] extracted from the *Bombxy mori* silkworm and were micro-molded into an array of MNs. The silk fibroin MNs has enough mechanical strength to pierce the model skin (porcine skin), and they have been shown to be suitable for encapsulating methylene blue used as a model drug. Other early works also developed MNs from maltose [70]. Olatunji et al. [71] prepared MNs from fish scales to achieve low cost as well as good biocompatibility. Polymers are also blended with other materials in the desired proportion to improve the drug release profile [72,73]. The drug transport behaviour from MN is another factor for its choice [74].

### 2.3. Various Design of MNs

As we analyse the key MN features, it is imperative to consider the designs of these MNs more carefully as they are important for the modelling purposes. There are, in fact, a number of special designs amongst the MNs depending on their applications, for instance, one may consider the pocketed solid MNs for the higher load of drugs [75] and hollow MNs with multiple output ports [76]. These MNs are slightly modified from the basic MNs types discussed above and extend the utilities of the MNs in different ways. The success of MNs products and fabrication process highly depends on the reliability of these MN’s design for the chosen application.

Several studies have adopted the tapered MN design for drug delivery. Olatunji et al. [71] modelled the force on MNs produced from fish scales using the tapered needle design with varying tip dimensions. The study focused on fabricating fish scale biopolymer based MNs having adequate mechanical strength so as to permit the penetration of MNs into the skin without fracture. The modelling strategy employed by them is discussed in Section 4.1. Using simulations, they investigated the design of MNs of different structures and different materials prior to fabrication. This reduced the time and cost, which would have been involved in designing a wide range of MNs and carrying out skin insertion tests or mechanical strength tests on them.

**Figure 3:**
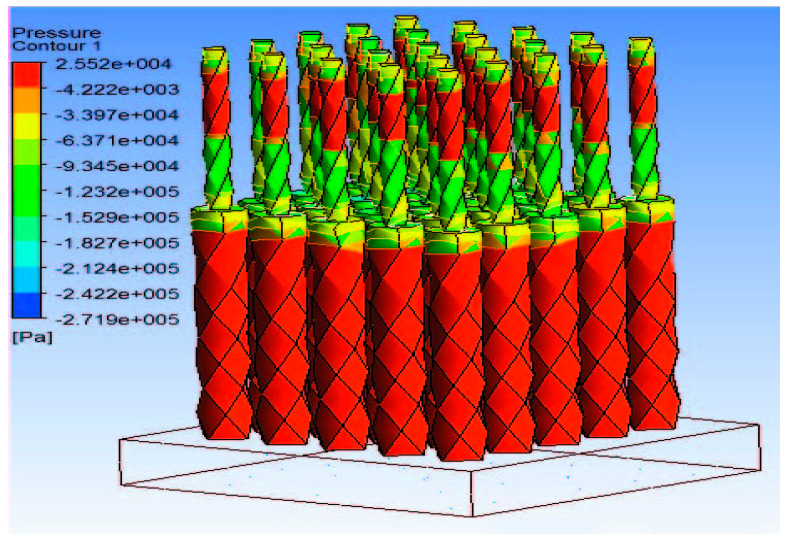
Pressure distribution along dual radii MN array (adopted from [79]).

Zhang et al. [77] employed the tapered design to model gene delivery into the body using a novel MN-based gene gun. They developed a model to represent the relationship between the delivery of microparticles using the MN-gene gun and MN parameters such as length and microparticle size. The tapered design has also been used to model amniotic fluid extraction for testing for Down syndrome in fetus using MNs [78]. By simulation analysis of various MNs and process parameters, the researchers were able to predict that longer MN lengths and insertion using vibratory action would result in more effective insertion into the skin and extraction of amniotic fluid. Simulation of pressure distribution along dual radii MN design has been used to investigate the fluid extraction using the tapered MN, as shown in Figure 3 [79]. The dual radius design whereby the upper part of the MN has a slightly higher radius than the lower part is applied to prevent the clogging of fluids, which is often associated with the small lumen of hollow MNs as a result of the micro nature of MNs.

**Figure 4:**
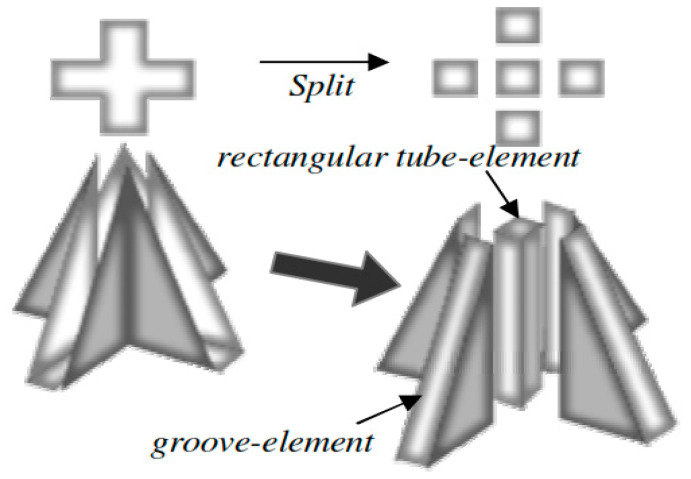
Two different capillary phenomena used in modelling of crown shaped MNs (adopted from [80]).

In an innovative approach to hollow MN design, crown-shaped MNs, which did not have an actual lumen but can be used for fluid extraction, were presented [80]. The quadruplets MNs, as they are referred to, were fabricated using X-ray lithography. This MN design is based on the generated capillary force between the nanosized slits that make up the boundaries of the MNs. This is demonstrated in Figure 4. A modified capillary model was derived for the quadruplet MNs since the typical capillary model for flow in a tube does not apply in this case. The predicted capillary height matched with experimental values.

**Figure 5:**
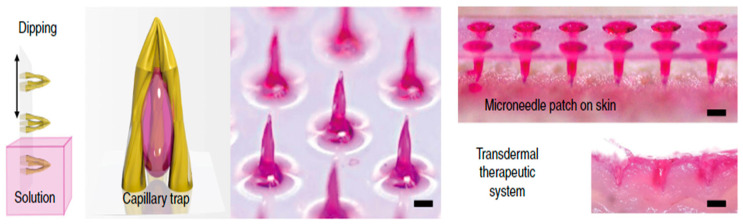
Merged-tip MN (adopted from [81]).

Lim et al. [81] have come up with a new design for use as a MN known as merged-tip MNs. These MNs are fabricated using photolithography using poly (ethylene glycol) diacrylate (PEG-DA) resin. MNs are dipped in a solution, and the solvent gets trapped in a solution by capillary force, as shown in Figure 5.

**Figure 6:**
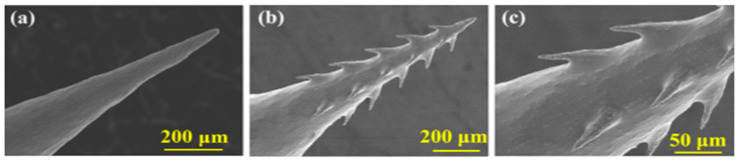
Images of the (**a**) parent MN, (**b**) bioinspired MN, and (**c**) barbs on the parent MN (adopted from [82]).

Recently, Chen et al. [82] have also fabricated MN geometry, which imitates a honeybee stinger using magneto-rheological drawing lithography. The authors designed microbarbs on the MN surface with the help of an ex-situ magnetic field (Figure 6). The authors subsequently performed modelling studies to show that the barbs reduced friction force, which facilitated an easy insertion of the MNs into the skin.

From the above discussion, it is evident that different MN structures have been fabricated by the researchers for efficient transfer of drugs into the skin. These systems are then considered at various bodies that approve MN based products, e.g., the US Food and Drug Administration [83]. For these approvals, controlling the variability of MN as a product during their manufacturing and achieve the desired clinical performance of these systems still remains as one of the key challenges. We believe the incorporation of the Quality by Design (QbD) concept into the MN manufacturing practice can be helpful in addressing this challenge [84]. This notion is supported by The International Council on Harmonisation of Technical Requirements for Pharmaceuticals for Human Use, which provides a framework for adopting a QbD approaches for designing and manufacturing of MNs [83].

In addressing the same issue of controlling variability of MN characteristics, the design of experiment (DoE) analysis was exploited by Jing et al. [85] for fabricating sharp tip silicon MN array by a dry etching process. Similarly, Held et al. [86] incorporated DoE while fabricating silicon MNs. Coupling QbD, DoE, and mathematical modelling and simulation studies with the typical steps for laboratory experiments and fabrication processes should certainly help in achieving improved quality of MNs.

## 3. MN Modelling Approaches

During the modelling of MNs, one needs to convert either the conceptual or the real MN design into a computational domain, which can be used to run numerical simulations or other modelling exercise (e.g., approaches using applied mathematics concepts using a direct solution of governing equations). It seems that the first work which reported MN modelling was in 1999 when the authors looked at modelling fluid extraction using hollow MNs [76]. These authors used numerical simulations to study the performance of micromachined MNs with channels for coupling flow. In general, it is observed that the mathematical models have been used mostly to verify the performance of the designed MNs [87]. After the computational domain is created, the properties of both the MNs and MNs treated skin need to be selected following the required conditions in the design stage. MN properties include the mechanical properties of the MN structures (e.g., Young’s modulus, Poisson’s ratio, ultimate tensile strength, etc.) for insertion studies [88] and properties of the loaded drug formulation determines the diffusion process. The skin properties (e.g., thickness, Young’s modulus, porosity, etc.) are also related to the permeation behaviour which will affect the diffusion of the drug molecules in the MNs treated skin [89] and the deformation of the skin due to MNs insertion [90,91,92]. We consider these points further as discussed in various sub-sections below.

### 3.1. Parameters for MN Modelling

**Figure 7:**
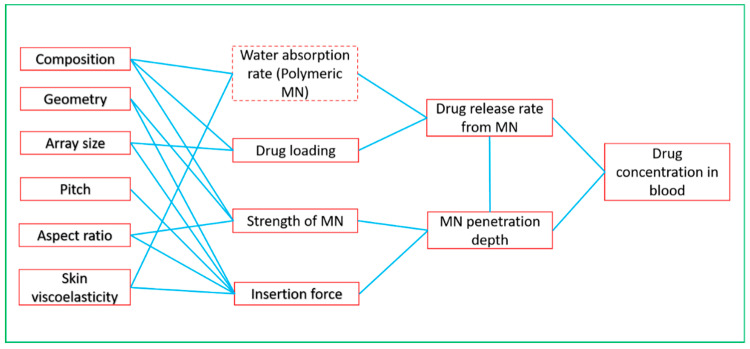
Relationship between skin and MN design parameters and therapeutic efficacy.

One of the main objectives of MN modelling is to obtain the required design parameters of MNs so that the desired/optimum MN performance can be achieved. The outputs of such modelling exercise are typically the penetration depth and drug release rate from MNs. These outputs are related to various parameters of both skin and MN [71,93]. The factors responsible for MN performance are its chemical compositions and geometric parameters such as needle height, tip-radius, base diameter, needle geometry, needle thickness, and needle density, etc. (Figure 7). The water absorption rate is also an influential criterion that should be kept in mind while modelling the dissolving and swellable MNs [94]. Skin viscoelasticity determines the amount of force required by MN patch to penetrate the skin [90]. To obtain effective improvement of skin permeability, effects of different MN tip radius, length, and number in an array have been modelled in previous studies [95,96,97,98,99,100,101,102]. Other researchers have focused on modelling the specific MN application method [103,104].

**Figure 8:**
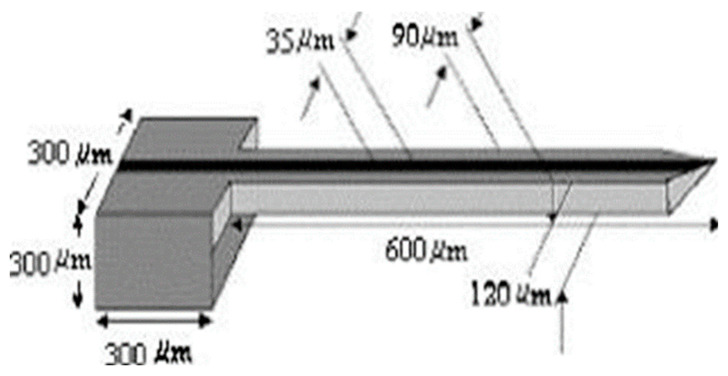
An example of a designed hollow MNs modelled (adopted from [105]).

The dimensions of the designed MNs need to be converted into input parameters for modelling, so the created computational domain is usable for further modelling and simulation exercises. The generated domain is a substitute for the designed MNs for the computer simulation where different tests can be achieved within that domain. For example, a computational domain of a hollow MN created in the computer program ‘Ansys’ is shown in Figure 8 [105].

The parameters involved to describe the MNs geometry are input to the computer program as well as the alignment of those MNs for a modelling process. For the parameters describing MNs’ geometry and arrangement on the MNs patch, there are five key aspects which have been analysed and discussed: the penetration depth/MNs length, both tip and base diameter of the MNs, center to center spacing between two MNs, numbers of MNs in the array and the distribution of the MNs in an array (square, diamond, triangle, rectangle or special design [106]). However, these parameters do not individually affect the drug diffusion rate, and they are connected as a synergetic system. Therefore, new parameters are introduced to define the relationships between these MNs parameters, thereby describing the system properly for the purpose of optimisation. For example, a parameter called aspect ratio (α) can be selected to define the relationship between two key parameters: (i) MN base radius ‘R’ and (ii) center to center spacing ‘P_t_’ (Pitch) [106]. α describes the ratio of the pitch over a MN base radius so the arrangement of the MNs on a patch can be confirmed according to the conditions that are limited in a MN design stage. Following the definition of MNs geometry and arrangement, other parameters need to be input accordingly for different purposes of the simulation. The parameters related to skin are also needed to be included for an effective diffusion study of the therapeutic using the MN [107]. In the above studies by Das and his group members, the partition coefficient for the drug at the skin/blood capillaries was not determined.

Skin composes of different layers (i.e., SC, viable epidermis (VE) and dermis (DE)), which have different mechanical strengths and molecular diffusivities values (Figure 2). Modelling of MN pierced skin, therefore, often focus on using different parameters that reflect the skin properties, e.g., skin insertion force and penetration depth. For instance, it has been suggested that solid MNs have a higher stiffness than hollow and dissolving MNs [71]. The insertion force is further related with MN tip angle and radius of tip’s curvature. We will discuss these aspects further in the latter part of the review.

Once the appropriate skin domain and their properties are chosen, the domain is used for simulation of drug permeation studies, which require information about the related drug molecules. Parameters of the molecules, such as the molecular weight and partition coefficient, can affect the drug diffusion coefficient. To demonstrate how molecular weight affects diffusion rates, Gomaa et al. [89] selected a series of six structurally related ionic xanthene dyes with a diverse range of molecular weights to run the simulation on MN treated porcine ear skin. The molecular weights of the six dyes ranged from 366.80 Da (Rh 110) to 10,000 Da (RITC-D). The results of this study confirmed that the molecular weight is one of the significant factors that affects the molecular diffusion rate in the skin in general, and MN pierced skin in specific.

### 3.2. MN Modelling Tools

**Figure 9:**
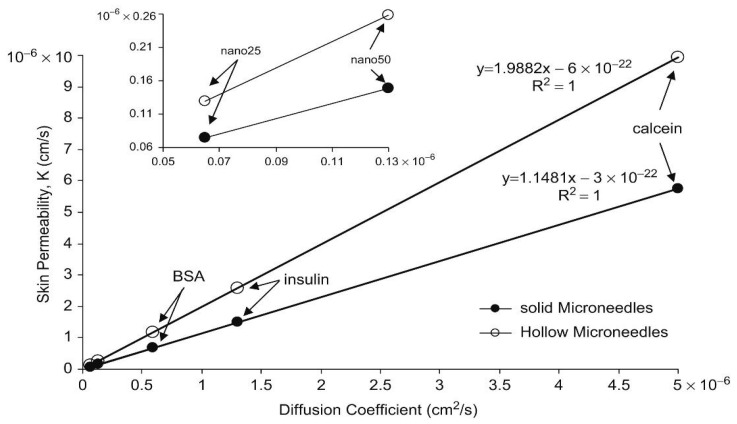
Relationship between the permeability and diffusion coefficient of the optimum solid (n = 20, R = 19 μm and A = 0.04 cm^2^) and hollow (n = 13, R = 140 μm and A = 0.53 cm^2^) MNs (adopted from [111]).

A number of computer-based tools have been developed for MN modelling using both in-house programming and commercial software. These tools are usually developed for specific modelling purposes. For instance, when the parameters such as the aspect ratio (α) were needed to be tested for the optimisation of the squared MNs patch, an in-house java programming tool was developed to achieve the purpose [106]. An optimum α was then determined so that the information may be used to optimise the physical dimensions of MNs with greater accuracy. Furthermore, based on the optimised parameters acquired from the program, the permeability of different drug molecules through MNs treated skin can be predicted by using relationships for the diffusion coefficient. For example, the correlations between diffusion coefficient and skin permeability of some sample molecules (Calcein, insulin, bovine serum albumin, nanosphere particles with radii of 25 and 50 nm, respectively) which were deduced from theoretical relationships are presented in Figure 9 [106].

**Figure 10:**
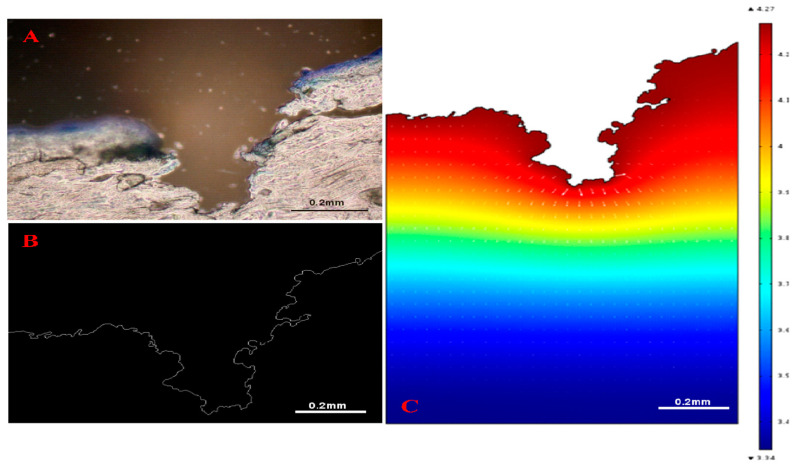
The histological image of sliced skin is processed by a MATLAB-based image processing tool. (**A**) the original image of skin histology; (**B**) the outline of the skin captured by the image processing tool and (**C**) simulated diffusion profile of insulin in MN-pierced skin (adopted from [108]).

**Figure 11:**
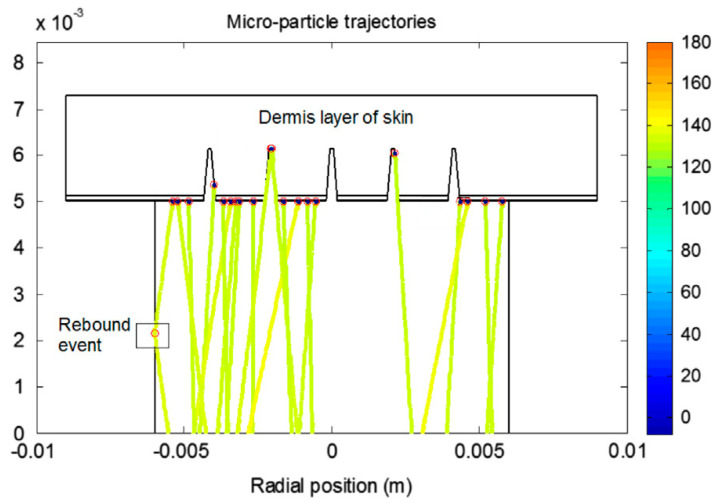
Micro-particle’s trajectories delivered by gene gun in MN pierced skin samples (adopted from [77]).

A relatively recent study used a MATLAB-based image processing tool to acquire skin pore profiles from histological images that show the cross-sectional views of MNs treated skin [107]. The computational domain in this study was directly obtained from the histological images of MN treated skin and used for computer simulations of drug delivery (Figure 10). Zhang et al. [77] have also developed a MATLAB-based tool which was used for simulating trajectories and penetration depth of micro-particles delivery by gene gun into MN-pierced skin. The objective of the study was to study computationally how MNs could reduce the skin resistance to particulate delivery in skin such as those encountered in gene guns. An example particle penetration profile of the micro-particles through the MN pierced skin layers is shown in Figure 11.

The diffusion profiles in the computational domain are generally simulated using commercial software that are based on a numerical method such as the finite element method (FEM), e.g., COMSOL [71,108], Ansys [105,109,110], Preview [90] and Abaqus [38]. After the designed models are imported into that software, the diffusion results can be determined following the configurations input by users. We discuss these points in more detail in the next section of this paper.

## 4. Case Studies on MN Simulation and Optimisation

This section highlights the key work done by various researchers in modelling MN-based TDD. The review is organized according to key aspect of MN based drug delivery addressed in the paper.

### 4.1. Simulation of MN Insertion Force and Effect of Skin Properties

As discussed above, various MNs have been developed in the past to allow for TDD. The particular MN designs rely on the manufacturing techniques used for fabrication, and their success is totally dependent upon their ability to puncture the skin [90]. However, the skin has a viscoelastic property, and therefore, this should be considered while carrying out modelling MN insertion behaviour. Sufficient force is required to ensure that the MNs are properly penetrated into the skin. Numerous studies have been focused on characterizing the MN insertion behaviour into the skin using various imaging methods and by performing MN penetration measurements using in vivo and ex vivo skin tissue [2,85,93,112,113]. Based on different experimental data and skin properties, researchers have now developed mathematical models to study the effective insertion force [112]. These models can predict the force required without the need of carrying out expensive and time-consuming insertion experiments.

Aoyagi et al. [114] carried out a FEM simulation study for hollow MNs and demonstrated that the maximum stress is encountered at the tip of the MN, and that the sharper the MN tip angle are, the easier it is for the MNs to penetrate the skin. Kong et al. [115] studied the implications of MN geometry and skin mechanical properties on the insertion force using FEM. They used tapered hollow metallic MN of different geometries (e.g., wall angle and thickness, tip area, etc.) to simulate the deformation and failure of skin tissue and the required insertion force of MN using different stiffness and failure stresses. A needle was inserted into the skin with uniform velocity of 1.1 mm/s under the assumption that this is a quasi-static process. The simulation results showed that the insertion force magnified linearly with enlargement of tip area of the MN and reduced with an increase in wall angle. It was also shown that the thickness of different layers of skin have a negligible effect on the insertion force [115].

**Figure 12:**
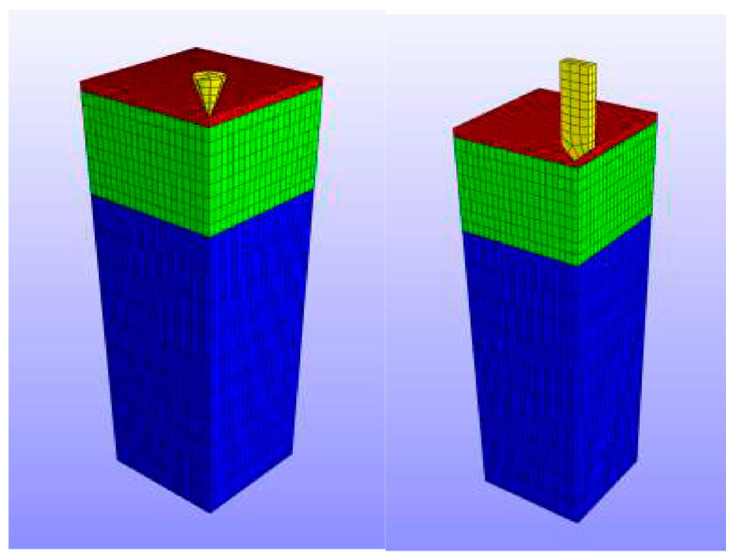
A numerical mesh used for finite element method (FEM) modelling of MN design (adopted from [90]).

As part of numerical modelling of MN systems, one is required to generate the specific numerical meshes of the computational domain over which the governing equations of the specific numerical model can be solved. The following two examples illustrate this point further. Groves [90] has analysed the mechanical behaviour of skin when the MNs are inserted into it. They have carried out a series of predicative FEM analyses of human skin behaviour, which incorporate the epidermis, dermis and hypodermis. Groves [90] used the Ogden model of hyperelasticity to determine the mechanical characteristics of all three skin layers when MNs are inserted. For the case of solid MNs, the simulation is mainly focused on the skin deformation during insertion rather than the deformation of the MNs themselves. Unlike Aoyagi et al. [114], the deformation of the solid MNs was ignored during the analysis of MNs insertion, and therefore, only the mechanical properties of skin have been used in the modelling. Figure 12 shows an image of the numerical mesh used by Groves [90] in their analyses. The average number of elements was 12000 in their numerical mesh and axial symmetry of the computational domain was assumed to reduce the computational time.

Boonma et al. [91] used fine triangular mesh to study the deformation of tissue and calibrate the magnitude of the stress tensor around the insertion site of MNs. In their mesh, only the deformation of skin was recorded, and the modified domain was then employed for conducting further numerical simulations [91].

**Figure 13:**
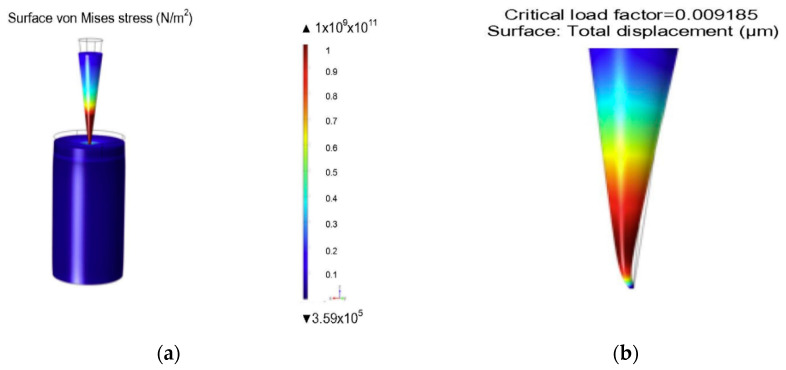
(**a**) Carboxymethylcellulose and maltose (CMC/MAL) dissolving MNs pierce skin sample under 5N load; (**b**) the buckling force is predicted based on the mechanical properties of the MNs (adopted from [88]).

The dissolving MNs consisting of polymers are relatively soft compared to the solid MNs (polymeric). Hence, the mechanical properties of dissolving MNs are imperative to be measured in order to optimise the formulation of the MNs and the maximum load it can bear. An example model [88] of dissolving MNs that consists of carboxymethylcellulose and maltose (CMC/MAL) is shown in Figure 13.

Olatunji et al. [112] analysed various combinations of forces acting during MN insertion in skin as given in Equation (1).

(1) $$F_{\mathrm{insertion}}=F_{\mathrm{bending}}+F_{\mathrm{indentation}}+F_{\mathrm{cutting}}+F_{\mathrm{buckling}}+F_{\mathrm{friction}}$$

where F_bending_ is the force that can bend the skin, F_indentation_ is the force that the MNs start disrupting the SC layer, F_cutting_ is the force that the MNs start piercing into the skin, F_buckling_ is the force causing skin deformation and F_friction_ is the frictional force during the MNs penetration. The forces were calculated for conical shaped MN using FEM and results were compared with experimental data. The results of those force components were shown to be not only dependent on the mechanical properties of the skin but also affected by the geometry and alignment of the MNs on the patch. The deformation of the skin caused by those forces could be different from the shape of the MNs, thereby changing the results of the simulation. Simulation results from the FEM agreed well with the experimental results carried out using neonatal porcine skin mounted on Franz diffusion apparatus.

Recently, Kim et al. [116] have developed a touch activated MN drug delivery patch and the authors have performed a modelling study to determine the effects of the applied force on the quantity of drug permeated from the MN into the skin. The relationships between normal force, quantity of drug release, wetting area of skin and quantity of drug permeated were expressed in the form of simple equations with a view to improve the user-friendliness of the developed approach.

From the above discussion, it is evident that most of the modelling for MN insertion in skin have been performed taking into account a single MN rather than the design of the whole MN array. This seems to limit the range of validity of these results because an accurate calibration of the insertion force should take into account both the individual MN design as well as the particular distribution of the individual MNs on the patch (e.g., square or rectangular distribution of the MN), e.g., to investigate the effect of MN interspacing (pitch width) on the insertion force.

### 4.2. Simulation of MN Enhanced TDD

Once a particular MN design is converted into a modelling domain (e.g., Figure 8) and suitable numerical meshes are created (e.g., Figure 12), the effective TDD study can be simulated based on parameters discussed in the previous section (e.g., Section 3.1; Figure 7). MNs create holes in the skin, which cause the drug molecules to penetrate the skin and reach the systemic circulation (Figure 2). These holes are more often chosen for simulation instead of the MNs model itself as the holes are directly connected to the skin. As a result, a complete simulation of TDD using the MNs can be considered as a system in which the MN is just one crucial component. The system conventionally consisted of three components: the MNs (or the holes they create), the skin and blood stream, which can be further extended (e.g., different skin layers) for higher model accuracy. For example, the three components system for pharmacokinetic study can be expressed as Equation (2) which defines the cells in skin as ‘bricks’ that blocks the drug molecules and reduce the diffusion rate [117]:

(2) $$V_{b}\frac{{\mathrm{dC}}_{b}}{\mathrm{dt}}=\left(\frac{\mathrm{dQ}}{\mathrm{dt}}\right)S_{a}-K_{e}C_{b}V_{b}$$

(3) $$V_{b}\frac{{\mathrm{dC}}_{b}}{\mathrm{dt}}=\left(\frac{\mathrm{dQ}}{\mathrm{dt}}\right)S_{a}-\left(K_{e}+K_{12}\right)C_{b}V_{b}+K_{21}C_{t}V_{t}$$

(4) $$V_{t}\frac{{\mathrm{dC}}_{t}}{\mathrm{dt}}=K_{12}C_{b}V_{b}-K_{21}C_{t}V_{t}$$

where K_12_ and K_21_ are the transfer rate between skin reservoir and blood circulation, C_t_ is the concentration in the skin reservoir and V_t_ is volume of distribution in the skin reservoir. Although the pharmacokinetic model can display the mass transfer status of all components in the system at both transient and steady states, the parameters such as K_12_, K_21_ and K_e_ are difficult to quantify. Therefore, the blood stream is sometimes assumed to have 100% absorption on drug solutions to avoid the complexity and back diffusion of the drug molecule into the skin is ignored [117].

Milewski et al. [118] developed a diffusion-compartmental mathematical model-based quantitative in vitro–in vivo correlation for drug transport across MN treated skin. The model considered two parallel permeation pathways (intact skin pathway (ISP) and microchannel pathway (MCP)), barrier-thickness-dependent diffusional resistance, microchannel closure kinetics, and drug pharmacokinetics. It was defined that all mass transfer resistance comes primarily from viable tissue (VT). The total flux (J_TOT_) was defined as [118]:

(5) $$J_{\mathrm{TOT}}=f_{\mathrm{ISP}}J_{\mathrm{ISP}}+f_{\mathrm{MCP}}J_{\mathrm{MCP}}$$

where f_ISP_ is the fractional skin surface area of the intact skin pathway, J_ISP_ is the flux through the intact skin pathway, f_MCP_ is the fractional skin surface area of the microchannel pathway, and J_MCP_ is the flux through the microchannel pathway. The cumulative amount-time profiles for skin layer (SC and viable tissue (VT)) were solved using Fick’s law of diffusion. In addition, they considered microchannel closure process which affect the drug transport in vivo. The permeability of the microchannel was interpreted with respect to the fractional skin surface area of the microchannel that remains open. The pore closure process affects the drug diffusion process and Equation (5) was modified by incorporation of new parameter for skin closure given by:

(6) $$J_{\mathrm{TOT}}=f_{\mathrm{ISP}}J_{\mathrm{ISP}}+f_{\mathrm{OMF}}f_{\mathrm{MCP}}J_{\mathrm{MCP}}$$

Here f_OMF_ stands for the open microchannel surface area fraction. The value of f_OMF_ is unity for the in vitro process and Equation (6) changes to Equation (5). The drug is taken away by the systemic circulation once it crosses the viable skin tissue. A regression analysis was performed on drug pharmacokinetic data which showed a good match with the microchannel closure rate and in vitro permeation data.

**Figure 14:**
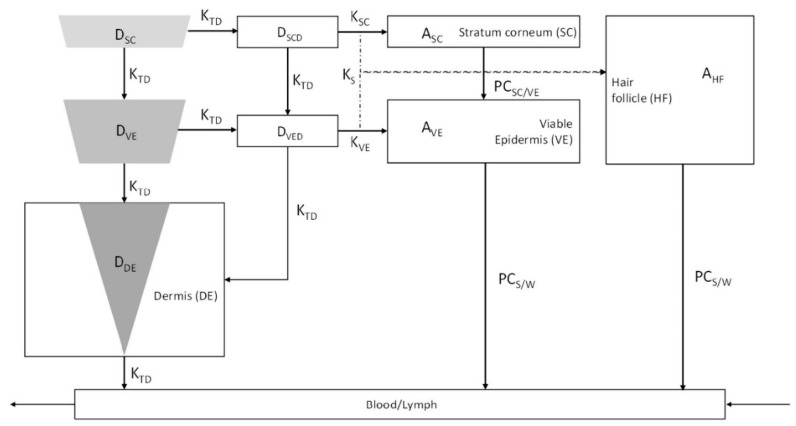
Drug release pathway from the MNs in the intradermal compartment reaching the blood and lymphatic circulation (adopted from [92]).

Rajoli et al. [92] developed a physiologically based pharmacokinetic (PBPK) model for modelling MN arrays containing cabotegravir and rilpivirine. The intradermal compartment is divided into four sub-compartments (i.e., SC, VE, DE and hair follicles (HF)) as shown in Figure 14. The value of different parameters indicated in Figure 14 are not discussed here and can be obtained from the paper Rajoli et al. [92]. The authors studied the effect of needle length, MN hole radius and release rate on the pharmacokinetics of rilpivirine. The model proved to be efficient in designing a novel formulation for chronic transdermal drug administration of the drugs.

### 4.3. Governing Equations for Drug Transport in MN Treated Skin

The skin layers perform as obstructions between the drug formulation in MNs and blood stream at different length scales (Figure 1). The diffusion of the drug molecules is therefore hindered, thereby, causing a concentration gradient in the skin. The diffusion profile of the drug molecules in the MNs treated skin is typically simulated using Fick’s law in which a diffusion coefficient is employed to relate the concentration variation within the skin with the diffusion flux. The steady and transient state of diffusion can apply Fick’s first (Equation (7)) and second laws (Equation (8)), respectively.

(7) $$J_{\mathrm{ss}}=-D\frac{\mathrm{dC}}{\mathrm{dx}}$$

(8) $$\frac{\partial C}{\partial t}=D\frac{\partial^{2}C}{\partial x^{2}}$$

Here J_ss_ is steady state flux, D is the diffusion coefficient, C is the drug concentration in the skin and x is the depth from top surface of the skin. The effective diffusion coefficient, which is related to the properties of the skin and size of the drug molecules, is approximated using Stokes–Einstein equation (Equation (9)) for drug transport through MN-pierced skin [119].

(9) $$D=\frac{k_{B}T}{6\pi \eta r}$$

Here, k_B_ is the Boltzmann’s constant, T is the absolute temperature, η is the viscosity of drug molecule and r is the radius of the drug molecules. McAllister et al. used Equation (9) to determine the drug diffusion coefficient in the drug solution within the holes created by the MNs. The authors then used a hindrance factor to work out the effective diffusion coefficient within skin. However, skin has different layers and complicated inner structures which are likely to make the values of effective diffusion coefficient calculated from this approach inaccurate.

Due to the special characteristics of skin, three other methods have been introduced in the literature in order to increase the accuracy of the calculations for drug diffusion coefficient as discussed below:

1. The diffusion coefficient can be deduced from the partition coefficient considering skin as a multilayer structure. The values of the diffusion coefficient are different for each layer of the skin, which can be calculated separately using Equation (10) [120].
  (10) $$D=-\frac{J_{\mathrm{ss}}h}{K_{i/j}c_{0}}$$
  Here, K_i/j_ is the partition coefficient between different diffusion compartments (donor compartment, SC, VE and DE), h is the thickness of the skin layer and c_0_ is the initial drug concentration in the skin layer.
2. To further explore the properties of different layers, the skin is considered as a porous material in which tortuous channels are defined to exist as pathways for the transportation of drug molecules. Equation (9) provides diffusion coefficients for every layer in the skin. While the drug molecules are inside those channels, the hindrance factor H(λ) also needs to be included where λ is the ratio of molecule radius over skin pore radius. Therefore, the size of the molecules decides the significance of the hindrance factor. Equation (11) shows the hindrance factor in cases where λ is less than 0.4 [121].
  (11) $$H\left(\lambda \right)=\left(1-\lambda^{2}\right)\left(1-2.104\lambda +2.09\lambda^{3}-0.095\lambda^{5}\right)$$
  After the hindrance factor is calculated, it is imported into Fick’s law along with the porosity and tortuosity of the skin which have been shown in Equations (12) and (13) [121,122].
  (12) $$J_{\mathrm{ss}}=-\frac{\varepsilon }{\tau }D^{\infty }H\left(\lambda \right)\frac{\mathrm{dC}}{\mathrm{dx}}$$
  (13) $$\frac{\partial C}{\partial t}=\frac{D^{\infty }H\left(\lambda \right)}{\tau^{2}}\frac{\partial^{2}C}{\partial x^{2}}$$
  where D^∞^ is the diffusion coefficient of the drug solution at infinite dilution, τ is the tortuosity of the channels and ε is the average porosity of the skin.
3. The third method aims to acquire the diffusion coefficient via experiment where the time lag (the time duration when the diffusion reaches its steady state) needs to be measured. Although this method considers the skin as homogeneous material, the diffusion coefficient is still more accurate than that obtained using Equation (6). The theoretical relation between the diffusion coefficient and the time lag is shown in Equation (14) [123]:
  (14) $$D=\frac{l^{2}}{6t_{\mathrm{lag}}}$$
  where t_lag_ is the time lag and l is the thickness of the skin. There are also other studies which consider factors such as back diffusion of the drug molecules in skin [124], however, they are not elaborated in this review.

Recently, Rzhevskiy et al. [125] have developed a correlation to determine the microporation enhanced transdermal drug flux. The equation is based on simplified assumptions and parameter approximation as shown in Equation (15).

(15) $$J\approx 0.36n_{p}r_{p}{\mathrm{MW}}^{-0.6}C_{v}$$

where C_v_, n_p_, r_p_ and MW are the vehicle drug concentration, skin pore density, pore radius and the molecular weight of the drug, respectively. The validity of the equation has been confirmed by a regression analysis of literature data. The equation has been further validated by performing in vitro experiments on human abdominal skin with three different drug molecules.

### 4.4. Simulations of TDD in MNs Treated Skin

The MNs act as the drug delivery vehicle in the system where all the alterable parameters are included. The parameters that are related to the geometry and alignment of MNs have been mentioned in Section 3. Those parameters must be combined with the MN types to complete the simulations. With a view to illustrate these points clearly, we divide the discussions according to the MN types. Modelling of swellable MNs is an area which has not been explored fully yet and needs further attention.

#### 4.4.1. Simulation of Solid MNs

The solid MNs can be either coated with drugs while insertion (coat and patch) or used to create pores on the skin where drugs will be applied after the MNs are removed (poke and patch). The poke with patch method has more capacity for drug loading, so the pores created by MNs should be considered as reservoir for drug solutions. The permeability (K) inside the reservoir can be described by [119].

(16) $$K=f\frac{D}{L}$$

where, f is the fractional area of the pore over the skin and L is the length of the pore. The shape of the pore created by MNs is related to the MNs’ geometry. To optimise the MNs enhanced diffusion in the system, the crucial factor is the arrangement of MNs on the patch. Based on the imported MNs model, there is a function (g) introduced to characterise the pores on a square patch and is given by [111].

(17) $$g=\frac{n^{2}R^{2}}{A}$$

where, f is the fractional area of the pore over the skin and L is the length of the pore. The shape of the pore created by MNs is related to the MNs’ geometry. To optimise the MNs enhanced diffusion in the system, the crucial factor is the arrangement of MNs on the patch. Another important condition is that the pitch P_t_ is defined as the center to center distance between two adjacent MNs. It ensures that the MNs on a patch do not overlap with others and avoid a disordered pattern [111].

(18) $$P_{t}=\frac{\sqrt{A}}{n}\geq \alpha R$$

where α is the aspect ratio of the pitch and must be greater than 2 to avoid overlap.

Equation (18) indicates the MNs on patch are arranged in square pattern. To achieve other patterns such as triangular, diamond or rectangular, the pitch component in transverse direction (P_tn_) and longitudinal direction (P_tm_) must be specified. After the pattern has been chosen, the optimised α will be deduced so that the relationship between pitch and the parameters in Equation (17) can be found. By applying Fick’s law, the correlation between the permeability and diffusion coefficient of different drug molecules based on those optimum parameters can be calculated which are shown in Table 1.

Uppuluri et al. [101] analysed the implications of different MN geometries (e.g., length, number of MNs and shape) on the TDD of rizatriptan drug molecules. A scaling analysis was carried out using the Buckingham π theorem, which defined the interplay among different dimensionless parameters of drug concentration and MN geometry as shown in Equation (19) [87].

(19) $$\frac{C_{t}}{C_{s}}=K^{{}^{\circ}}{\left[\frac{S_{a}L^{4}K_{e}}{V_{b}\mathrm{hD}}\right]}^{n}$$

where C_t_ and C_s_ represent the drug concentrations in blood and MN, respectively, K° is a dimensionless constant, n is unknown power, S_a_ is the surface area of MN patch, K_e_ is an elimination rate constant, h is the thickness of skin, L is MN length, V_b_ is volume of receptor fluids (in vitro) and D is the diffusion coefficient of drug in untreated skin. Correlations for dimensionless concentrations with different dimensionless parameters of MNs are as shown in Equations (20) and (21).

(20) $$\frac{C_{t}}{C_{s}}=4.864\times 10^{-3}{\left[\frac{h}{L}\right]}^{-1.592}\;\;\;\;\;\mathrm{for}\;0.8\leq \frac{h}{L}\leq 2$$

(21) $$\frac{C_{t}}{C_{s}}=0.224{\left[\frac{S_{a}}{L^{2}}\right]}^{-0.798}\;\;\;\;\mathrm{for}\;78.5\leq \frac{S_{a}}{L^{2}}\leq 491.5$$

Good correlations were observed between the dimensionless concentrations determined theoretically as well as experimentally [101]. 

Lyashko et al. [126] developed an analytical approach for modelling and optimisation of drug concentration for solid MN treated skin. They considered the MNs as point sources of drug concentration and solved a convection-diffusion equation to work out the concentration of drug as functions of time and distance. The paper also tried to investigate the existence of a unique solution for mathematical problems for Fick’s law with the chosen initial and boundary conditions and provided the proof of the existence of the mathematical solution.

Numerical simulations have also been performed for solid MNs coated with drug formulations. Römgens et al. [38] performed computational modelling to find the optimum geometry of MN array based on the number of antigens presenting cells, which were defined to depend on the induced immune response. Three-dimensional FEM was done on Abaqus describing the diffusion and kinetics of antigen transfer into the skin. The set of governing equations was solved to study the effects of various MN parameters (MN array interspacing, length, base radius, drug loading and release rate) on the number of activated cells in both the epidermis and dermis. The simulations could identify the optimum values for center-to-center distance of MNs for the applied drug dose. Also, it was shown that the length of the MN influenced the immune response with base radius and release rate causing minimal effect.

#### 4.4.2. Simulation of Hollow MNs

**Figure 15:**
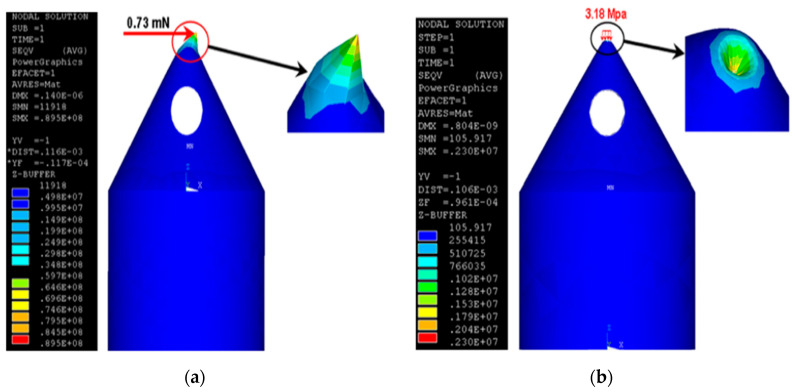
(**a**) Bending stress analysis and (**b**) axial stress analysis (adopted from [109]).

Hollow MNs have narrow capillaries that can infuse drug solution into the skin. Various numerical simulations have been completed on flow and structural analysis of MNs. Shibata et al. [127] performed experimental and simulation studies to determine the mechanical stability of hollow MNs made from silicon dioxide. Bodhale et al. [109] performed structural and computational fluid dynamics (CFD) analyses of hollow side-open polymeric MNs for TDD application. They proposed a hollow MN with both cylindrical and conical part with holes on the side of MN. The simulation results for axial and bending stresses are shown in Figure 15. The simulation results showed minimum deflection of tip due to applied pressure at the tip. The CFD analysis was also carried out to determine the pressure distribution and flow rate of the fluid flowing through the hollow MNs. Different pressure ranging from 10–100 kPa was applied on MN based on the MN pump specifications and flow was analysed. The numerical results showed that the pressure and velocity distribution remained uniform in the MN array which is desirable to deliver the drug in proper proportion. The proposed design was shown to be suitable for integrating with micropumping device for delivery of drug.

**Figure 16:**
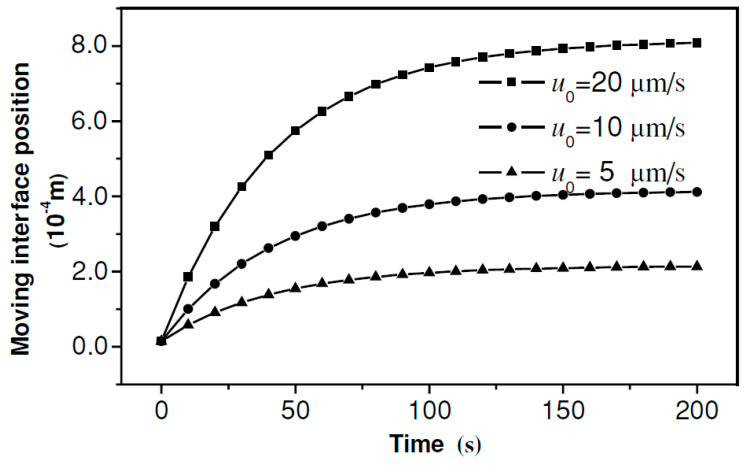
Influence of injection velocity on the position of the moving interface (ε = 0.2, φ = 0.01 m^3^ liquid/m^3^ tissue, β = 0.005 m^3^ liquid/m^3^ tissue/s) (adopted from [128]).

The hollow MNs have channels that can continuously deliver drug solution into the skin. However, the annular wall of the MNs limits the contact area of the drug solution to the skin, hence, the parameter f in Equation (16) needs to be redefined which is shown in Equation (22) [106].

(22) $$f=n\pi \frac{{\left(R+W\right)}^{2}-R^{2}}{A}$$

where W is the annular gap width of the hollow MNs. The drug delivery mechanism of hollow MNs is more complicated compared to the solid MNs because the drug delivery rate of hollow MNs is not only relying on the diffusion but also depends on the injection process. Therefore, a concept of ‘moving interface’ is introduced to describe the boundary that separates saturated and unsaturated skin tissues. The velocity of the moving interface should be higher during the MN injection than passive diffusion rate as given below [128]:

(23) $$u_{\mathrm{int}}=\frac{u_{0}\varepsilon }{\varepsilon +\left(1-\varepsilon \right)}e^{-\frac{\beta t}{\varepsilon +\varphi \left(1-\varepsilon \right)}}$$

where u_int_ is the velocity of the moving interface, u_0_ is initial injection velocity, φ is the drug solution absorption rate per unit volume of tissue, β is the absorption coefficient of drug taken by blood stream and ε is the average porosity of the skin. The results indicate that higher diffusion rate can be achieved by increasing u_0_ while the porosity and absorption rate of skin are considered as intrinsic variables. In the said study, the moving interface position was measured in an assumed thickness x_0_ =15 µm skin sample which is shown in Figure 16.

Recently, Liu et al. [129] developed a model to study the relationship between the drug delivery rate through hollow MNs and skin resistance pressure. They proposed a physically based model for calculating the driving force for drug infusion and verified it through experiments. The resistance to the drug delivery rate was estimated using a pressure loss calculation. The final expression for the overall pressure balance is given in Equation (24).

(24) $$\frac{F-F_{f}}{{\pi D}_{s}^{2}/4}=\frac{8{\rho Q}^{2}\left(t\right)}{\pi^{2}}\left(\frac{1}{N^{2}D_{\mathrm{mn}}^{4}}+\frac{{\lambda L}_{t}}{D_{t}^{5}}+\frac{2.02}{N^{2}D_{\mathrm{mn}}^{4}}+\frac{\zeta_{1}}{D_{\mathrm{mn}1}^{4}}+\frac{\zeta_{2}}{{\mathrm{ND}}_{\mathrm{mn}}^{4}}+\frac{\zeta }{D_{t}^{4}}\right)+\frac{128{\mu L}_{\mathrm{mn}}}{{\pi D}_{\mathrm{mn}}^{4}}\frac{Q\left(t\right)}{N}$$

In Equation (24), F is the force applied by the pump on the syringe, N is the number of MNs and F_f_ is friction force between the piston seal ring and syringe wall. All of the terms included in the equations are defined in Table 2. The values of the parameters are also included as they provide an excellent indication of the significance of these parameters for MNs treated skin. The authors concluded that the drug delivery rate was mainly influenced by a resistance pressure. Liquid also tends to backflow when the flow rate is increased to a certain value.

**Figure 17:**
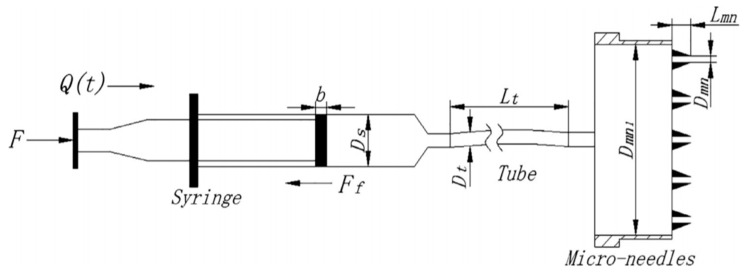
Physical model of syringe driven MN delivery system (adopted from [129]).

Micropumps have been investigated in combination with hollow MNs to carry out the drug delivery and diagnosis. Chen et al. [130] proposed an expansion model to theoretically characterise the drug delivery rate using a hollow MN array driven by micropumps. They assumed that the infusion through MN causes ‘spherical expansion’ and a ‘spherical diffusion’ in the tissue due to relatively high fluid pressure in the MNs. The authors performed experiments on silicon rubber and polyacrylamide gel, and the results from these experiments qualitatively agree with the analytic results from the model for drug delivery. Similarly, Haldkar et al. [110] performed a modelling study on hollow MNs integrated with micropumps for biosensor application (Figure 17). The paper analysed the effect of the shapes of hollow MNs on the flow of liquid inside the micropumps. The MNs were modelled for various pressure differences using FEM to achieve the required flow for biosensing. It was concluded that pentagonal shape of MN is the best for achieving the desired mass of fluid at the biosensor location.

#### 4.4.3. Simulation of Dissolving MNs

Dissolving MNs are of utmost importance now for TDD as they can improve regulatory compliance and safety for patients. The dissolving MNs have similar mechanisms to solid MNs when using poke with patch method. However, the drug matrix in the reservoir of dissolving MNs is formulated so that it can be released over time. Therefore, the actual amount of drug solution is a time dependent function that is related to the dissolving rate of the drug matrix, i.e., the volume change rate of a conical dissolving MNs is given by [131].

(25) $$\frac{{\mathrm{dv}}_{c}}{\mathrm{dt}}=-\pi \frac{k_{D}}{\rho }\left(\frac{\tan\;\theta \;}{\cos\;\theta \;}\right)h^{2}\left[C_{s}-\left(\frac{1-\beta }{\beta }\right)C\right]$$

where, v_c_ is the volume of the dissolving MNs, k_D_ is dissolution rate constant of the drug matrix, ρ is the density of the drug matrix, h is the height of the MNs, C_s_ is the solubility of the drug matrix in water, C is the drug concentration in the skin, β is the mass fraction of drug in the MNs and tanθ is the original ratio of radius over the height of MNs. Equation (23) indicates the boundary conditions of the simulation are changing with time and highly related to the dissolution rate of the drug formula. Besides, the alignment of the MNs and mass fraction of the drug in the MNs is also adjustable parameters that can affect the diffusion rate.

This approach was further investigated by Ronnander et al. [94] for modelling the transport of sumatriptan from pyramidal shaped dissolving MN. They studied the effect of drug loading, needle height, and pitch width on the release profile of sumatriptan in the skin. The change in drug concentration in the skin with time was determined as follows [94].

(26) $$\frac{\mathrm{dc}\left(t\right)}{\mathrm{dt}}=-\left(K_{L}c\right)+4\left[\frac{k_{D}\tan\;\theta \;}{\rho \cos\;\theta \;}\right]h^{2}\left[\frac{\beta \rho -c}{v_{\left(t\right)}}\right]\left[c_{s}-\left(\frac{1-\beta }{\beta }\right)c\left(t\right)\right]$$

where, K_L_ is elimination rate constant and v(t) is volume of skin layer. The parameters K_D_ and K_L_ were estimated by carrying out the nonlinear regression of experimental data. Cumulative percentage of drug release M(t) is calculated by using the equation:

(27) $$M\left(t\right)=1-\frac{d_{s}P_{w}^{2}\left(c_{\mathrm{SD}}\left(t\right)+c\left(t\right)\right)}{\left({\beta \rho v}_{c,0}+m_{0}\right)}$$

where P_w_ is pitch width, C_SD_ is drug concentration in solid phase, v_c,0_ is the initial volume of skin layer, d_s_ is skin depth and m_0_ is mass of drug in the baseplate. The results showed that drug concentration in the skin increased with increase in drug loading and height of MN. As the pitch width was increased the level of sumatriptan permeation in the skin also reduced.

Ronnander et al. [132] reported a model which was developed to evaluate the efficacy of sumatriptan administration. The model simulated the dissolution of pyramidal shaped MNs and the diffusion of sumatriptan. The authors used the model for simulating three different cases of sumatriptan DMN formulations and concluded that by reducing the MN pitch width significantly one could increase the sumatriptan diffusion in the skin, in line with the findings of Kim et al. [131]. Such modelling studies assist in optimal design of MNs in terms of drug loading and MN array geometry. Recently, Zoudani et al. [133] performed a set of simulations for dissolution of conical shaped polymeric MN in porous medium. They used the approach proposed by Kim et al. [131] to model the dissolution kinetics and study the effects of drug loading and pitch width (Equation (25)). Zoudani et al. [133] introduced the concept of hindrance factor to invesgate the effect of drug molecular radius on the drug effective diffusion coefficient in the skin, which was not considered by Ronnander et al. [132] (Equation (11)). Their results showed that faster dissolution is achievable by increasing the initial drug loading and no significant changes were seen on varying the pitch size [133].

Both Kim et al. [131] and Ronnander et al. [132] have considered the dissolution of MN as a linear process (e.g., constant height to base radius ratio throughout the dissolution process), which may not describe well the actual changes in the shape of MN [134]. The height of the MN decreases at a faster rate as compared to the base radius making the value of tanθ (Equation (27)) a variable which should be accounted for in Equations (25) and (26). Also, the mathematical modelling involved lot of input parameters which makes it difficult for non-experts such as medical and pharmaceutical simulation with other MN dissolution experiments. Chavoshi et al. [135] had earlier reported a mathematical model which was developed on the basis of autocatalytic effects within the MN polymer. The autocatalytic dissolution effect was incorporated in the model to predict the drug release profiles in skin. The authors used conical shaped MNs and the drug release rate was modelled as a function of polymer matrix degradation rate of poly (lactic-co-glycolic acid) (PLGA). The main point of the MN is the hydrolysis reaction occurring between steric bonds of the polymer which results in a reduction of the molecular weight and consequent degradation of the MN array. The molecular weight of the polymer changes with the water and polymer concentration was defined as in Equation (28).

(28) $$\frac{M_{n}}{M_{n}^{0}}=\exp\left(-k\left[C_{H_{2}O}\right]\left[C_{\mathrm{PLGA}}\right]t\right)$$

In Equation (29), M_n_^0^ is the initial number averaged molecular weight while k is the degradation rate constant. The mass conservation equations of the model were solved for all system components using a finite difference scheme, and the model solutions were validated against experimental data from the literature [136]. This modelling approach uses concentration term for the water content within the MN which is difficult to determine because both the mass and volume of the MN change with time. Therefore, it would be reasonable to explore an alternative approach where the total mass of water would be the measurable quantity. Secondly, while the authors assumed an effective shape of the MN in their paper, they ignored the concept of effective diffusivity of the drug in the skin, such as that discussed by Davidson et al. [107].

Watanabe et al. [104] have explored the implications of MN array geometries on drug permeability in skin. On the other hand, Chen et al. [102] have performed a scaling analysis of insulin loaded polymeric MNs to study the effect MN geometric parameters on MN penetration efficiency. Based on Equation (20), the following correlations were developed between the dimensionless insulin concentration against different dimensionless parameters considering that all other variables remained unchanged. Equations (29) and (30) show the relation between dimensionless insulin concentration and different length of MNs. This analysis was based on the research results of Uppuluri et al. [101], which were discussed earlier.

(29) $$\frac{M_{n}}{M_{n}^{0}}=\exp\left(-k\left[C_{H_{2}O}\right]\left[C_{\mathrm{PLGA}}\right]t\right)\;\;\;\;\;\;\mathrm{for}\;0.29\leq \frac{h}{L}\leq 0.80$$

(30) $$\frac{C_{t}}{C_{s}}=1.2293{\left[\frac{S_{a}}{L^{2}}\right]}^{-0.113}\;\;\;\;\;\;\mathrm{for}\;32.65\leq \frac{S_{a}}{L^{2}}\leq 256$$

The correlations indicated that the effect of the change in MN length on the effective insulin delivery can be easily explained by optimal scaling analysis. This enabled Chen et al. [102] to demonstrate that MN length is a significant factor in improving TDD of insulin, interpreted due to an enhancement of the drug diffusivity coupled with a reduction in the drug penetration time. Amodwala et al. [137] performed a statistical optimisation on polymeric MNs composition to achieve maximum needle strength. The axial needle fracture force analysis was performed for MN patch with different composition ratio of polyvinyl alcohol and polyvinylpyrrolidone. They statistically analysed the factor-response relationship and provided an optimised MN formulation.

Besides these, new concepts are emerging based on the previous parameters to increase the accuracy of modelling and simulation, i.e., effective thickness is introduced which is calculated from the effective permeability of VE using Fick’s law. It is considering the skin deformation after the MNs insertion in order to give a more realistic value to skin thickness because the MNs reduce the distance between the drug loads to the bloodstream. The effective thickness H_eff_ can be calculated using Equation (31) [107].

(31) $$H_{\mathrm{eff}}=\frac{D_{\mathrm{VS}}C_{m}}{J_{\mathrm{ss}}}$$

where D_VS_ is the diffusion coefficient in the VE and C_m_ is the concentration coated on the MNs.

To apply all these parameters for simulation, the MNs design needs to be digitalised for computer program to process. Finite element method (FEM) is chosen as the algorism by most of the studies because it can provide detailed diffusion or force distribution profiles [91,138] and the time range allow to apply transient model or steady state [139]. The MNs based simulation is a way to test the efficiency and feasibility of the MNs design apart from the experimental method. The advantages of the simulations are that they can predict the diffusion results before the experiment. The accuracy of the diffusion results relies on the parameters defined at the modelling stage so the theoretical data of those parameters are crucial and if they are well measured, the prediction will be more convincing when compared with the experimental results.

Furthermore, the simulation of drug delivery using dissolving MNs can be advanced by modelling the drug-polymer interactions within the MN structure. This allows the inclusion of the mechanism by which the drug travels through the MN structure and the distribution of the drugs within the MN. This advances from the previous assumption of the skin-MN interface acting as a bulk constant concentration.

**Figure 18:**
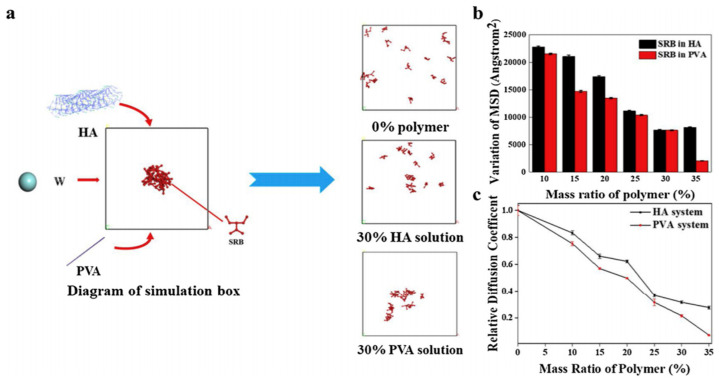
(**a**) Sketch to illustrate difference in diffusion behaviour of Sulforhodamine in different polymer solutions and (**b**) the variation of mean square displacement (MSD) mean square displacement (MSD) for Sulforhodamine (SRB) and (**c**) the relative diffusion coefficient of SRB (adopted from [140]).

Feng et al. [140] used molecular dynamic simulation based on the quantum chemical calculations to model the binding energy and electronegativity differences in the materials used for MN fabrications and the loaded drug. For this study, polyvinyl alcohol (PVA) and hyaluronic acid (HA) blends were used for the MN fabrication while the model drug was Sulforhodamine (SRB). The system was allowed to perform simulations of 50,000 steps to achieve thermal equilibrium. The last 50,000 frames were also analysed for calculating diffusion coefficient and deviation in mean square displacement (MSD) of SRB beads. Figure 18 indicates that the deviation of MDS for SRB beads in PVA solution is smaller than that of HA solution when the mass ratio is between 10% ∼ 35%. The simulations indicated that the drug preference to diffuse in the HA as compared to the PVA enabled the concentration of the loaded drug at the tips of the MNs. The simulation result was validated with experimental results. This allows for more efficient drug delivery and minimal wastage of loaded drugs within the MNs. This is particularly important for highly potent drugs that are required in small quantity and are also costly.

## 5. Conclusions

MN modelling is still at its early stage but its importance in the development and optimisation of MN systems are growing as evidenced from an increasing number of publications as well as a complex range of issues being modelled by these publications. Given the interests in these models, it is concluded from this review that development of mathematical models of MN systems will enable efficient design and optimisation of these systems, as the system themselves are developed for more complex range of TDD problems. As the theoretical basis of MN-based TDD continues to be developed further, the MNs modelling will be a key in developing the MNs for researches and manufactures.

Modelling and optimisations have already been performed on different types of MNs. Different algorithms have been developed for solving the series of equations, thus, providing frameworks which can be adapted to predict performance of future MN arrays. Furthermore, the role of skin has also been incorporated in the modelling to understand the MN-based TDD process in an efficient way. The optimised MNs have been shown to enhance the permeation of drug potentially in many laboratory studies. However, there are yet to be sufficiently accurate and robust MN models for practical, medical or manufacturing applications. Most of the modelling studies have been performed considering the single MN which makes it hard to incorporate the combined effect of the whole MN array. Also, full attention needs to be placed on the material properties of the MNs for maintaining the pharmacokinetics and pharmacodynamics of drug molecule. Some models ignore the skin deformation and changing boundary conditions as there is a continuous change in contact between the microneedle and skin with time. Neglecting these factors while modelling can result in under or overpredictions of the MN performance and, consequently, the result can be unreliable.

With more information acquired from MN experiments in vivo and in vitro, the interactions between the MNs and the skin as well as the drug diffusion profiles in the skin can be resolved. This will solve various challenges faced during preclinical and clinical evaluation of novel formulations and modes of delivery. MN modelling are therefore expected to play a greater part in improving the fabrication and application of MN systems in the future.

## Figures and Tables

**Table 1:** Correlations of permeability (K) of drug molecules for different patterns of MNs patch. K is related to the diffusion coefficient (D) as discussed by Al-Qallaf and Das [111].

MN Distribution in a Patch	Permeability (cm/s)
Square	K = 1.6185 × D − 0.0008
Diamond	K = 0.8125 × D − 0.0029
Triangular	K = 0.936 × D − 0.0007
Rectangular	K = 1.622 × D − 0.0002

**Table 2:** Constants and variables used in Equation (24) [129].

Constants	Definition	Values	Variables	Definition	Unit
Dmn	Inner diameter of MN	$1.8\times 10^{-4}\;m$	Q(t)	Volumetric flow rate of liquid drug	m^3^/s
Dmn1	Inner diameter of micro-needle cavity	$1.1\times 10^{-2}\;m$	F	Force that the pump push block applies on syringe	[N]
Ds	Inner diameter of syringe	$8.4\times 10^{-3}\;m$	ζ	Friction coefficient at syringe outlet	-
Dt	Inner diameter of soft tube	$1.4\times 10^{-3}\;m$	ζ 1	Friction coefficient at connection from soft tube to micro-needle cavity	-
Lmn	Length of MN	$9\times 10^{-3}\;m$	ζ 2	Friction coefficient at connection from MN cavity to needle	-
Ρ	Density of liquid drug	1000 Kg/m^3^	Ff	Friction force between seal ring of piston and syringe wall	[N]
Μ	Dynamic viscosity of liquid drug	$1.005\times 10^{-3}\;\mathrm{Pa}\cdot s$	λ	Friction factor	-
N	Number of MNs	5			
